# Is the Race Degenerating?

**Published:** 1876-06

**Authors:** John Murray

**Affiliations:** Rochester, Pa.


### ARTICLE IV.

### *Is The Race Degenerating?*

BY JOHN MURRAY, D. D. S., ROCHESTER, PA.

Solomon might have occasion, if he were with us now, to administer the same reproof to us that he did to a numerous class in his day, to whom he said: "Say not thou, what is the cause that the former days were better than these? for thou dost not enquire wisely concerning this."

There perhaps was never a period in the past history of our race when some mal contents were not found to assume that the former times were better than the present, or that the race had degenerated morally, mentally, or physically.

It is comparatively of recent date that this class of fault-finders has assumed the garb of science. Formerly, they were found among the ignorant and uninformed, but latterly, they appear among the admirers of science, and supported such names as Prosper Lucas and Charles Darwin, so that now to make the charge that the race is degenerating, smacks of science. Darwin accounts for the improvement in the brute creation by the theory of natural selection, which is the highway to perfection, but leaves the human race when they reach that elevated plain to be governed by other laws than physical force, to drift to imbecility and decay.

The doctrine of evolution and hereditary transmission is very frequently introduced in our dental associations by some of our most worthy and esteemed members. And it is sometimes assumed that about all the dental maladies that our race is heir to, come upon us by hereditary transmission and maternal impressions.

That there is some foundation, in fact, for the doctrine of hereditary transmission, will be freely admitted; but there is a possibility of carrying this view beyond the bounds of truth, and thus obstructing the true channels of knowledge.

I was present at a dental convention once, and heard a gentleman, whom I very much esteem and respect, relat the following fact: He said a young lady of his acquaintance lost the lower portion of the right leg by a railroad accident. The lost member was supplied by a well adjusted substitute, so much so, that few would notice the loss by her walk. In course of time she was married, and when her first born was presented, it was minus the same member. This was thought to be a remarkable circumstance, and yet if the doctrine be true, it is not at all remarkable: it is only what we might expect, and we would be warranted in expecting every other child born by the same mother to be brought forth in the same condition : and if the children born to this mother were females, we would much more confidently expect *their* children to be born in the same condition.

I know a much more remarkable case myself, because it has no maternal impression or hereditary transmission to help us out of the difficulty. I knew a child born without the right hand by a mother who was perfect in all her limbs, and she in turn bore a large family of children who were all perfect. With one whole generation to perfect the hereditary taint, it is strange that none of the children took after the mother. In the case of the first lady named, if the maternal impression was so prompt and responsive, now that the habit is established, might we not expect in a few generations that nature would supply a substitute for the defective part, resembling cork, or wood and leather combined and adjusted to the defective limb that the physical deformity might not be much felt.

If this hereditary transmission were as uniform and marked as its friends and advocates would have us believe it is, what a vast variety would we find in the physical conformation of the human family. The advocates of this theory contend that man is of great antiquity, and yet all these thousands, it may be millions of years, have not materially changed him. With a little variation in stature and complexion, and in his social habits, we find him everywhere, and in all ages, about the same.

But I have allowed myself to drift from my original design. I commenced with the purpose to show that our race had not degenerated, or at least that degeneration was not the result of hereditary transmission, but simply local and incidental causes that may depend upon climate and food.

The race has not degenerated in regard to longevity. Since the abbreviation of human life, men live about as long as in former years. They may not be as robust, or able to endure such hard-ships as their fathers or generations that preceded them. But this may be accounted for on the ground that they have not had the physical training.

We know how much may be accomplished by physical training in preparing men to endure hardships and fatigue. It must be remembered, in this age we are subject to more physical ills than men were in former ages, or else diseases are called by new and additional names.

In the bills of mortality, we are informed that many children die in infancy ; yet many deaths are recorded of persons who had reached the utmost limits proposed by inspiration, and some even surpass an hundred years.

Men of this generation are as large, and are as quick in their movements, are as athletic, and, with proper training, can endure fatigue and hardship as well as men could at any previous period. The Egyptians of the present time are as long as the mummies who are embalmed because of their prowess and victories fifteen hundred or two thousand years ago. Englishmen are larger now than were those of ancient times. It is said there cannot be found in the whole world a thousand coats of armor which a regiment of her Majesty's soldiers could put on. That there is no shrinkage in the human organism is shown in this—that the swords of the ancients are useless now because the hilts are too small to admit the hand of our modern warriors.

There is no proof found, either in pictures, skeletons or statues, that ever the human race was larger or more perfect than now. The acrobats of to-day can perform all the feats for which the Greek athletes were celebrated. A regiment of English, Russian, German or American soldiers would be more than a match for any equal number of any race that ever lived in any age, when armed with the same weapons, and equally familiar with their use.

Twelve centuries ago India and China were what they are now, no better and no worse. John Chinaman, as represented on the porcelain before the birth of Christ, presents no better physical developments than he does now. The Anglo-Saxon race, without any advantages of climate, and with the disadvantages of a luxurious living, has increased more largely than any other

Races may degenerate and become effeminate, but if the cause be abandoned, the system possesses an elasticity that will rebound to its normal state. Of physical degeneracy, without a change of food and climate, there cannot be found a well authenticated trace. All the clamor that is raised about the degeneracy of the race, results from discontents and an unwillingness to be satisfied with the present state of things.

Boynton and Webb swam across the channel between England and France—an aquatic feat that no Greek swimmer ever has, or ever could have, accomplished.

It is not true that whole races degenerate, any more than that they advance, although it is true that there are families or classes of the race that may degenerate, or are now degenerating, but they are those whose fathers and forefathers, through a long line of succession, have been given to intemperance and gross sensual indulgence. But this would not invalidate the argument that degeneracy does not take place when the people are of sober and virtuous habits, and are under the same conditions of food and climate.

Whatever would tend to physical degeneracy must also tend to mental imbecility. The physical cannot enjoy a state of advanced vitality, and the mind at the same time deteriorate ; body and mind are too intimately connected for such a process.

All that has been said against the theory of man's physical degeneracy may be said with equal truth against the idea of any mental deterioration of the race compared with the ancients. No century of age has witnessed such wonderful advancements in useful knowledge as the present. We stand and look with astonishment at the wonderful skill displayed in the Egyptian Pyramids, and heathen temples and ancient mounds ; but while we admire the architectural skill of these grand monuments, we fail to comprehend the design. So far as we can see, the works were aimless ; the benefit of the race had never entered into the conception. But what wonderful skill, founded on the idea of benefiting the race, has been displayed in the century in which we live! Art and science have advanced more than in the thousand years that preceded it. Thought is made to travel with the speed of lightning, and men are enabled to converse with each other on opposite hemispheres. So also we travel by machinery, propelled by steam with almost incredible velocity. The system of railroads, and the rapid communication of thought between man and his fellow man, separated by thousands of miles, has a direct tendency to spread knowledge and to elevate the status of human civilization. It is a fact worthy of the present century, that all the great discoveries in the arts and sciences have been prosecuted and consummated with a direct view to the good of the race.

We read of Demosthenes and Cicero, and justly admire them as great in their day; but we have had their superiors in Clay, Webster, and other orators of the age. Our statesmen, our orators and our generals of the present age are unequaled by any of the past ages. Even Solomon himself, stripped of the direct gifts of inspiration, has had, and now has, his peers.

If the race shows no signs of degeneration, mental or physical, as we think it has been shown, we must look for some other cause for the loss of so many teeth among American citizens.—*Pennsylvania Journal.*

